# Are we leaving someone behind? A critical discourse analysis on the understanding of public participation among people with experiences of participatory research

**DOI:** 10.1371/journal.pone.0273727

**Published:** 2022-09-02

**Authors:** Constanza Jacques-Aviñó, Elena Roel, Laura Medina-Perucha, Jasmine McGhie, Mariona Pons-Vigués, Enriqueta Pujol-Ribera, Irene Turiel, Anna Berenguera

**Affiliations:** 1 Fundació Institut Universitari d’Investigació en Atenció Primària Jordi Gol (IDIAPJGol), Barcelona, Spain; 2 Universitat Autònoma de Barcelona, Campus de la UAB, Plaça Cívica, Bellaterra, Cerdanyola del Vallès, Spain; 3 SerMas, Servicio de Salud, Centro de Salud de Potes, Villaverde, Madrid, Spain; 4 Servei Català de la Salut (CatSalut), Travessera de les Corts, Edifici Olímpia, Barcelona, Spain; 5 Departament d’Infermeria. Facultat d’Infermeria, Universitat de Girona, Girona, Spain; 6 SESPA, Servicio de Salud, Área I Atención Primaria, Asturias, Spain; Universitat Autonoma de Barcelona, SPAIN

## Abstract

Participatory research (PR) is on the rise. In Spain, PR is scarce in the field of health, although there is an increasing interest in the matter. A comprehensive understanding of the meanings and practical implications of “public participation” is essential to promote participation in health research. The aim of the study is to explore the discursive positions on PR among individuals with experience in participatory processes in different areas and how this understanding translates into practice. We conducted a critical discourse analysis of 21 individuals with experience in PR and participatory processes (13 women, 8 men), mainly from the field of health and other areas of knowledge. Sixteen were Spanish and the rest were from the United Kingdom (3), United States (1), and Canada (1). Interviews were conducted in person or by telephone. The fieldwork was conducted between March 2019 and November 2019. The dominant discourses on public participation are situated along two axes situated on a continuum: the purpose of public participation and how power should be distributed in public participation processes. The first is instrumental public participation, which sees participatory research as a tool to improve research results and focuses on institutional interests and power-decision making is hold by researchers and institutions. The second, is transformative public participation, with a focus on social change and an equitable sharing of decision-making power between the public and researchers. All discursive positions stated that they do not carry out specific strategies to include the most socially disadvantaged individuals or groups. A shift in the scientific approach about knowledge, along with time and resources, are required to move towards a more balanced power distribution in the processes involving the public.

## 1. Introduction

Participatory research (PR) implies that anyone affected by or interested in a phenomenon can be part of a research project and have some level of influence on the decisions of interest related to the topic [1]. Including the population in the research bridges the gap between academia and the public, and allows for research to focus on people’s needs. A fundamental aspect that is inherent to the concept of PR abides in its value from a democratic and ethical perspective, related to values such as social justice and autonomy [2]. PR recognizes the rights of individuals to have access and to make decisions in research processes that arise from the data they have provided and/or that may affect them [1]. At the same time, from a citizen’s viewpoint, it is important to reframe research as a process that belongs to the public, as research is mainly funded through public resources [3]. In addition, there is a great deal of evidence that community involvement has a positive impact on your health [4]. On the other hand, in recent years, social values towards research have changed, with studies suggesting a growing feeling of distrust towards research institutions and a greater desire to engage in participatory processes and to have access to information. Reasons for this change include increased mobility of individuals, access to new technologies and increased education [5].

To date, although PR is a growing phenomenon, several labels are used to refer to PR approaches, such as citizen science, public participation in scientific research, or civic science [6]. The use of different terms to refer to PR reveals different understandings of what is PR, and the definition and goals of PR are often ambiguous and vary across stakeholders, cultures, and countries. In addition, the role of the public is often unclear [3]. On the one hand, some PR processes engage the public in a rather superficial way, without truly involving the public in research decision-making [7]. On the other hand, some authors argue that the public’s input should be given the same importance as that of other partners in the research team and, therefore, should be included in all the steps of the research process, from study conception to results dissemination [8]. Within these co-production models of research (focusing on co-leadership, mutual learning, and shared benefits between science and society), reciprocity is a core principle [3]. For example, community-based participatory research (CBPR) is a research approach that aims to equally engage communities in all the steps of a research process in order to promote social changes that benefit these communities [9]. These different views of PR are reflective of a tension between a dominant scientific paradigm, which gives more value to the knowledge of academics, versus a CBPR paradigm, which is more egalitarian. This debate has been well described by E. Trickett, who posed a dichotomy between a utilitarian versus broad capacity-building worldview model [10]. For instance, there are examples of participatory processes conducted by administrations and organisations that focus on costs and benefits, as well as on the desire of improving their institutional image towards citizens rather than on social change [5].

Moreover, awareness of the potential benefits of PR is not uniformly distributed across research disciplines, with this approach being less commonly adopted in biomedicine compared to other domains [11]. In Spain, although experiences in participatory processes are scarce in health research, there is a positive predisposition and an increasing interest in PR, especially in the field of primary healthcare. However, there is a lack of training and knowledge about how to conduct participatory processes, and researchers have often doubts about who should be included, how to select participants, and how to take into consideration the potential conflicts of interest between the public and the research team [12]. Since researchers’ attitudes determine how research is conceptualized and developed, we believe that a comprehensive understanding of the meanings and practical implications of “public participation” for experts in PR is crucial to promote public participation in health research in our country [13]. Furthermore, PR has also been postulated as a tool to reduce social inequalities in health [14]. The key in research participation resides in the researchers’ attitudes that determine how, who explain research is conceptualized and developed [13]. Therefore, understanding the views of participation experts towards participatory processes could also provide valuable information regarding how inequalities are addressed in such processes.

Therefore, the aim of this study is to explore the discursive positions on PR among individuals with experience in participatory processes in different areas and how these understandings translate into practice (i.g., how PR or participatory processes are carried out), with a particular focus on Spain. These results will allow us to deepen and reflect on the mechanisms that facilitate or hinder participatory processes and will contribute to the development of PR processes in the field of health research in our context.

## 2. Methods

### 2.1 Study design

This is a qualitative study based on a Critical Discourse Analysis (CDA). Since we are particularly interested in how social inequalities are addressed in participatory processes, we adopted a CDA approach in order to gain insights into the ideological assumptions that are hidden in the discourses related to the values of participation [15]. The importance of carrying out a CDA lies in understanding discourse as a form of social action; considering the relationship between language, ideology and power [16]. The main principles of CDA are that social problems need to be addressed, discourse analysis is interpretative and explanatory [17]. As researchers, we are committed to produce knowledge contributing to processes of social transformation and to strive against social inequities in health. We believe this is a historical opportunity to understand that scientific knowledge cannot be exclusively conceived from a technical-scientific perspective.

### 2.2 Sampling and recruitment

The study population comprised 21 individuals with experience in PR or participatory processes. People were selected through purposive sampling [18]. Participants included renowned experts (researchers, activist and/or policy developers) who have published extensively or who are recognised by their peers to have extensive expertise. To compare experiences from Spain with those of countries with a longer trajectory in participatory processes, we included informants from the United Kingdom (UK), Canada and the United States (US). We mainly included informants working in the health field and particularly related to patients’ involvements and community health. To ensure discourse variation we also included informants with experience in other areas. They were invited to participate by e-mail or direct contact. Interviews were conducted in person or by telephone depending on the informants’ availability. Six other individuals were contacted but could not take part (they did not reply to the e-mail inviting them to the study). The characteristics of the informants are available in Table 1. Thirteen participants were women and 12 had experiences in health research.

**Table 1:** Informants’ characteristics who have experience in PR or participatory processes and who were interviewed.

Informant ID	Gender	Area	Country
I1	Woman	Health	Spain
I2	Woman	Health	Spain
I3	Man	Health	UK
I4	Woman	Education/ /Children’s participation	Spain
I5	Man	Housing/Gentrification	Spain
I6	Man	Environmental sciences/Health	Spain
I7	Man	Education/ youth participation	Spain
I8	Man	Environmental sciences	Spain
I9	Man	Environmental sciences/Health	Spain
I10	Woman	Environmental sciences	US
I11	Woman	Health	Canada
I12	Woman	Housing	Spain
I13	Woman	Environmental sciences	Spain
I14	Woman	Health	UK
I15	Woman	Social sciences / Feminism	Spain
I16	Man	Education	Spain
I17	Woman	Health	Spain
I18	Woman	Health	Spain
I19	Woman	Health	Spain
I20	Woman	Health	UK
I21	Man	Health	Spain

### 2.3 Data collection

We conducted semi-structured interviews between March 2019 and November 2019. These lasted between 30 and 60 minutes. At the beginning of the interview, participants were informed about the study purpose, reassured them of confidentiality of information divulged. All interviews were audio-recorded and transcribed verbatim. Fourteen interviews were done in person. Seven were telephone interviews. The interviews were conducted following a topic guide with open-ended questions focused on exploring the meaning and experience in participatory processes at all stages, including potential facilitators and barriers to such processes, strategies for public recruitment/contact and results and evaluation. We also explored their motivations and aspirations, their roles in participatory processes, views on people who participate, lessons gained from involvement and how inequities axes influence participation.

### 2.4 Data analyses

Data were analysed using CDA [17, 19, 20], to reach an in-depth understanding of how the experience and discourses on “*public participation”* have an impact on the hierarchical positions within participatory processes in the context of the research.

We conducted the analysis in several stages. Firstly, we read all the interviews repeatedly. Secondly, we identified discourses on the concept of *participation in research* within the transcripts, focusing on how these discourses may impact research practices. In the third stage, we considered the identified discourses in relation to how knowledge (academic vs lay) is validated. The next step was to reflect on whether current discourses on *participation in research* served a social purpose. In this stage, we focused on how discourses could sustain power relations and domination in research, especially regarding the relationship between researchers and the public. In the last stage, the analysis focused on the ways in which dominant discourses are challenged and resisted in some social spheres. Analyses was limited to social practices of analysis, not focusing on elements of text analysis (e.g., grammar) or discourse practice (e.g., text production) [21]. Procedures for data analyses were discussed by team researchers during several meetings.

Research quality and rigour was ensured using the criteria for assessing qualitative research [22, 23]. Triangulation procedures and reflexivity practices were other main procedures to ensure research quality [22, 23].

#### Ethical considerations

This study was approved by the Ethics Research Committee of IDIAPJGol (19/207-P). The processing of this data was carried out in compliance with Regulation (EU) 2016/679 of the European Parliament and of the Council of 27 April 2016 on the protection of individuals with regard to the processing of personal data and on the free movement of such data and Organic Law 3/2018. All informants gave their informed verbal and/or written consent prior to their voluntary participation. Oral consent was given in the telephone interviews, where people gave their consent to be recorded. Written consent was given in the face-to-face interviews. The verbatims were codified to maintain confidentiality. No financial or material compensation were offered to informants.

## 3. Results

**Fig 1:**
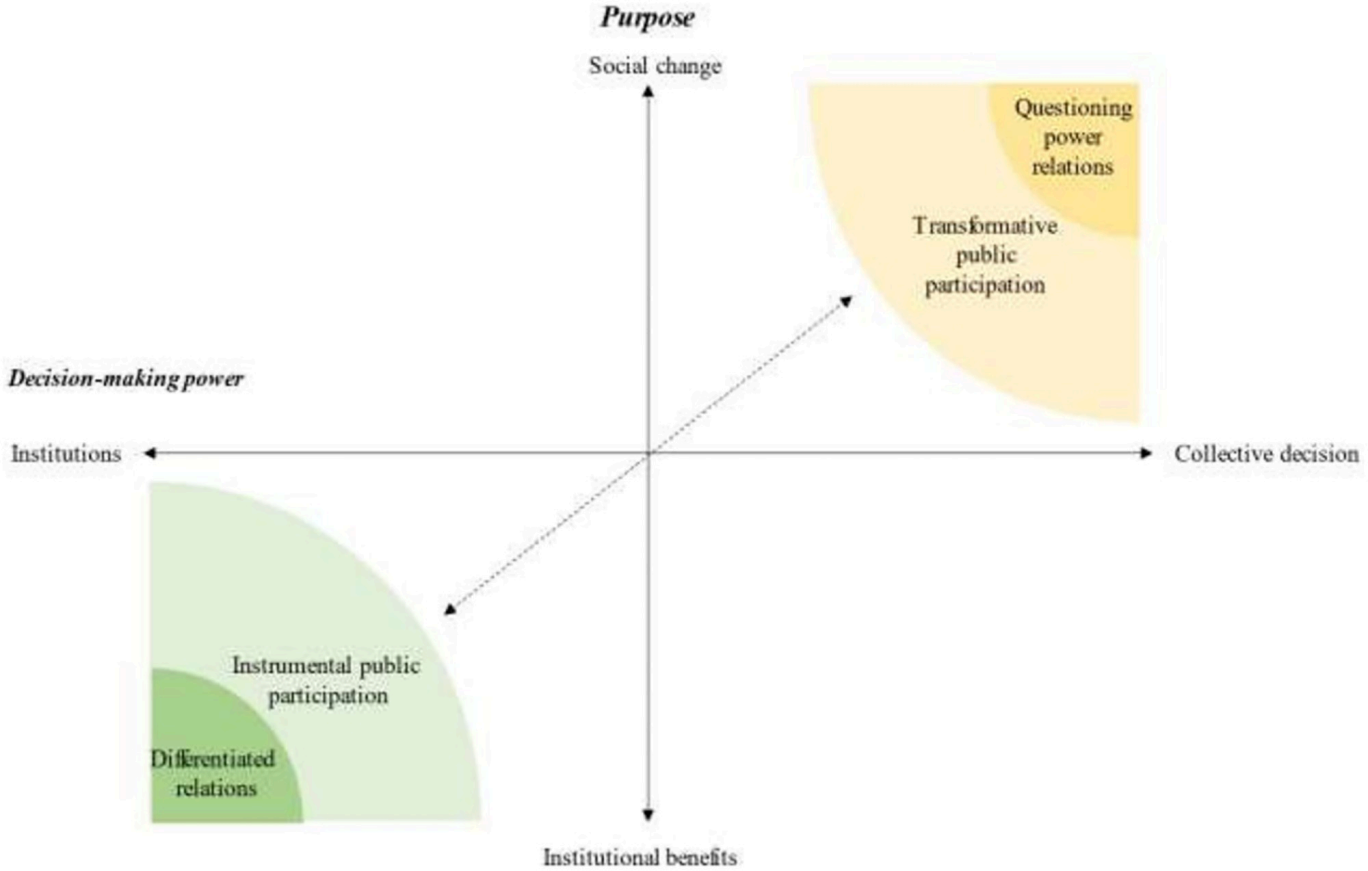
Discursive positions on PR according to a critical discourse analysis of people with experience in participatory processes (21 individuals).

We identified two dominant discourses among experts in participatory processes that can be placed at two opposite ends of a continuum defined by the purpose and the (theoretical) power distribution in PR (see Fig 1): 1) instrumental public participation (IPP), which views PR as a tool to improve research outcomes and is centred on institutional interests; and 2) transformative public participation (TPP), which advocates that PR should be transformative and focused on social change. Informants from the academia setting were generally closer to IPP views (especially those from Spain), whereas informants with experience in social activism were closer to TPP.

Dominant discourses on public participation IPP and TPP are placed on two axes: the purpose of public participation and how power should be distributed within public participation processes (Fig 1). Attaining social change and satisfying institutional benefits (e.g., obtain funding) are at the two poles of the “purpose” axis. The first pole implies changing the living conditions of the participants and promoting empowerment; the second pole implies a more pragmatic approach. Collective decision making and institutional power (e.g., political, scientific or economic organizations) are at the two extremes in the “power distribution” axis. The collective decision-making challenges power relations meaning that the research question and the interest in carrying out a project comes from the population concerned. The design and direction of the research are determined by the public. At the other extreme, research design and leadership rely on the institution conducting the research.

### 3.1 Instrumental public participation

In this discursive position, PR was framed as a tool to generate scientific knowledge closer to the public realities. The research outputs were placed at the core of participatory processes. PR was defined as a collaborative process in which the researchers and the public exchange their knowledge. According to the informant’s views, research does not always need to be participatory, as other mechanisms to generate knowledge may be required based on the nature of research. Similarly, the level of involvement of the public (e.g., from collecting data to designing and leading a research project) is also determined by the nature and aims of the project. Informants holding this discursive position, however, described mostly experiences in which the public’s role was limited to data collection, although they also highlighted the importance of disseminating study results to the public. Processes in which the public have a more prominent role (e.g., participating in research design stages) were perceived as more challenging.

“*I am a researcher*, *there are limits*, *I need to publish*, *I have more or less clear what I want to do and why*… *therefore explaining all those things to the citizens*, *and that citizens propose ideas and alternatives to solve something*, *personally*, *I have always doubted it*. *It is very nice*, …, *it sells well*, *but then*, *when it comes to implementing that in a real action or in some thing*, *it gets complicated*. *And in research we don’t have the money to work on those things calmly*.”

(I6)

Public contributions to research were seen as to *help* researchers and PR was perceived to be mostly beneficial to science and institutions. For example, PR enables researchers to collect often neglected data (e.g., patients’ experiences when interacting with the health care system); and/or large amounts of data (e.g., citizens themselves collect s data, related to the topics of interest of researchers through the use of mobile phones). IPP approaches included both quantitative and qualitative research projects. However, some informants from the field of quantitative research expressed concerns about the quality of the data collected by the public and emphasized that such type of data should be validated. Other concerns on the matter were the public’s rights to access and use the data, and how to achieve transparency and openness.

“*The main difficulty is finding the balance between scientific quality and reaching out to the widest possible public*. *(…) If you emphasize a lot on scientific quality*, *then you apply methods or use tools that nobody has and nobody is going to participate*. *If you emphasize on too much that everything being is worth it and allowing everybody to participate*, *then*, *you run the risk… sometimes not*, *but you run the risk that… of your project would not being scientifically valid*.”

*(I7)*

Despite the fact that participation was conceived as an exchange between two counterparts, in practice the roles of “the researchers” and “the public” were hierarchical. Informants holding an IPP discourse maintained the distinctive roles of *us* (i.e., researcher, with an active role) and *them* (i.e., the public, rather a passive role) and a more conventional power distribution in research. Expressions such as *we gave them* were commonly used. The public’s experiences and knowledge did not play a substantial role in the design of research processes. Training was commonly discussed as required for “the public” to be involved in research; whereas *expertise* in PR was thought to be gained through *experience* for “the researchers”. Increasing the scientific culture among the public was seen as an additional benefit of participatory processes. Conversely, attaining social transformation was not on the agenda and the public’s empowerment was mostly overlooked. Informants emphasized, however, that individuals taking part in participatory processes must perceive some benefits to remain engaged in the process.

The inclusion of disadvantaged populations (e.g., economically disadvantaged populations, the LGTBIQ+ community, children and the elderly, people with disabilities, etc.) was not at the core of IPP and was not a relevant matter for researchers holding this position. Some argued that the inclusion of minorities should only be targeted depending on the research topic. In practice, socially excluded communities were rarely engaged in participatory research.

“*I think a lot of PR does tend to recruit certain kinds of people usually well educated*, *usually not very ethnically diverse*, *probably more well-off*.”

(I14)

Identified barriers to IPP models were mainly the lack of funding and time, as well as the reluctance of some sectors of the scientific academia. In that sense, PR was perceived as a relatively new research tool that needs time to find its place within the scientific community. These barriers seemed to be more prominent and problematic in Spain, where PR is not as established as in other contexts such as in the UK, where a stronger structure for PR was mentioned to be in place.

“*(*…*) a lot of the difficulty is in the attitudes of colleagues who don’t see the need for it or think it’s a problem or gets in the way of research*, *of doing good research*. *So*, *there’re all kinds of problems to do with funding and resources*. *To do it properly you need to be funded properly*, *people need to know why they are doing it*”.

*(I16)*

The evaluation of participatory processes in research was structured in the same way traditional science is but it was also perceived as challenging. Evaluations focused on quantitative outcomes and in terms of benefits for research outputs, researchers and institutions.

### 3.2 Transformative public participation

In this discursive position, PR was seen as a key tool to promote and support social transformation and public’s empowerment. Informants holding this position placed the public at the core of research and participatory processes; and challenged the historical and hegemonic power held by institutions (e.g., white academic researchers as opposed to indigenous communities in America). PR was presented as not only beneficial but indispensable to generate transformative processes.

“*One of the ideas behind citizen science is empowering people so that they have more knowledge to make decisions*.”

(I4)

Informants were critical towards science not serving the public’s interests and highlighted that science has an historical debt with disadvantaged populations. Within that discourse, data was valued as a tool to raise awareness about matters of concern for the public.

“*Science is the quest for truth*. *So*… *maybe I am not interested in the quest for truth about social determinants because*… *(*…*) I mean I already know that being poor and whatever is going to impact on health*, *not to do more studies about that*. *Perhaps the quest for truth for*… *what a citizen wants is a transformation of reality*”.

(I21)

The TPP approach was constructed as an opposition to conventional PR models in which the public’s role is usually limited to data collection. Furthermore, informants within this position were critical with tokenistic forms of participatory processes that infantilize the public and minimize the value of lay knowledge. According to their views, such participatory processes tend to be manipulative processes designed to legitimize researchers and/or institutional interests.

“*If I* [researcher] *invite someone to participate in something because it is in my own interest*, *this is not participation*. *I could call it*, *in a very extreme scenario*, *manipulation*, *exploitation*.”

(I13)

“*Often the institution perceives and generates* [participatory] *spaces as ways of legitimating policies that they have already decided*.”

(I5)

One of the consequences of tokenistic participatory processes that was highlighted is the loss of trust from the public, that ends up questioning the legitimacy of the researchers’ motivations.

“*(*…*) there is always*, *and for a good reason*, *the perception that the academy is very utilitarian*, *manipulative sometimes*, *and in any case*, *selfish*. *The objective is writing the paper*, *the objective is depositing the thesis*, *and we are the raw material*.”

(I5)

In order to attain social transformation, power needs to be distributed so that the public can be greatly involved in all stages of research and decision-making processes. This position challenged established research structures by blurring the lines drawn between the roles of the researcher and the researched, individual roles becomes flexible and changed over time. The terms *experience* and *expertise* were used distinctively and framed as equally important. In that sense, hierarchies in TPP were diffused and even opposite to those of conventional science. Experiences of TPP included processes that were initiated by “the public” and, occasionally, led by them. The concept of “training” was replaced by the idea of “self-training”, which was necessary for all counterparts involved in the process and not only.

“*(*…*) [participation] can mean a lot of different things like I think that can mean more of an advisor role all the way up to a more true partnership or equal partnership um you know and even that other end of the spectrum where potentially members of the public are leading projects with more support from researchers (*…*)*”.

*(I11)*

Those informants holding a TPP discourse stated that equity should be a core value of participatory processes, which should include people who belong to disadvantaged populations. However, based on the informants’ narratives, it was noted that acting towards the inclusion of such social groups in PR was rather an ideal than a reality in practice. Informants did not report structured strategies in this regard, other than making additional efforts to reach out to these social groups (e.g., contacting an association representing an ethnic minority). However, it was pointed out that framing research processes without considering the intersection of social inequities enhances inequities within the research process itself. Remunerating participants was also seen as useful and ethical to increase diversity in participatory processes. Time and lack of resources (both for the public and researchers), as well as the lack of specific training for researchers, were the most common barriers mentioned for the inclusion of disadvantaged groups.

“*My experience (*…*) has been that people who participate are usually middle-class*, *for example in specific neighbourhoods*, *so participation is really white in these places*, *right*? *where these processes could be sometimes done in neighbourhoods with a lot of migration*, *but they* [research processes] *are formatted in a way that only one type of profile is convened*”.

(I17)

Lack of funding also emerged as one of the major barriers for PR, as well as academia’s reluctance. There was some concern about how funding itself hinders achieving an equal distribution of power among all counterparts. Failing to achieve social transformation, in the sense that citizens would refuse to collaborate in processes that do not benefit them, was also pointed out as a barrier itself.

“*In practical terms universities and research teams are quite bureaucratic*, *quite hierarchical*, *organisations*: *they exist in a competitive environment where resources are scarce and so on and so forth*, *they work with certain rules all of which make it very difficult to do meaningful public involvement*….”

(I3)

Interestingly, informants who maintained a TPP discourse were rather ambivalent regarding the success of public participation in practice. According to them, the structural and practical barriers that they had encountered greatly defined their PR experiences, and they rarely attained the desired predetermined aims (e.g., significant social change and transformation). Similar to IPP, evaluating participatory processes was challenging and usually not performed systematically. In this case, the public empowerment was seen as a potential evaluation indicator. Other informants mentioned self-reflection during and at the end of the participatory processes as very necessary for the evaluation.

### 3.3 The distribution of power in public participation, from theory to practice

**Fig 2:**
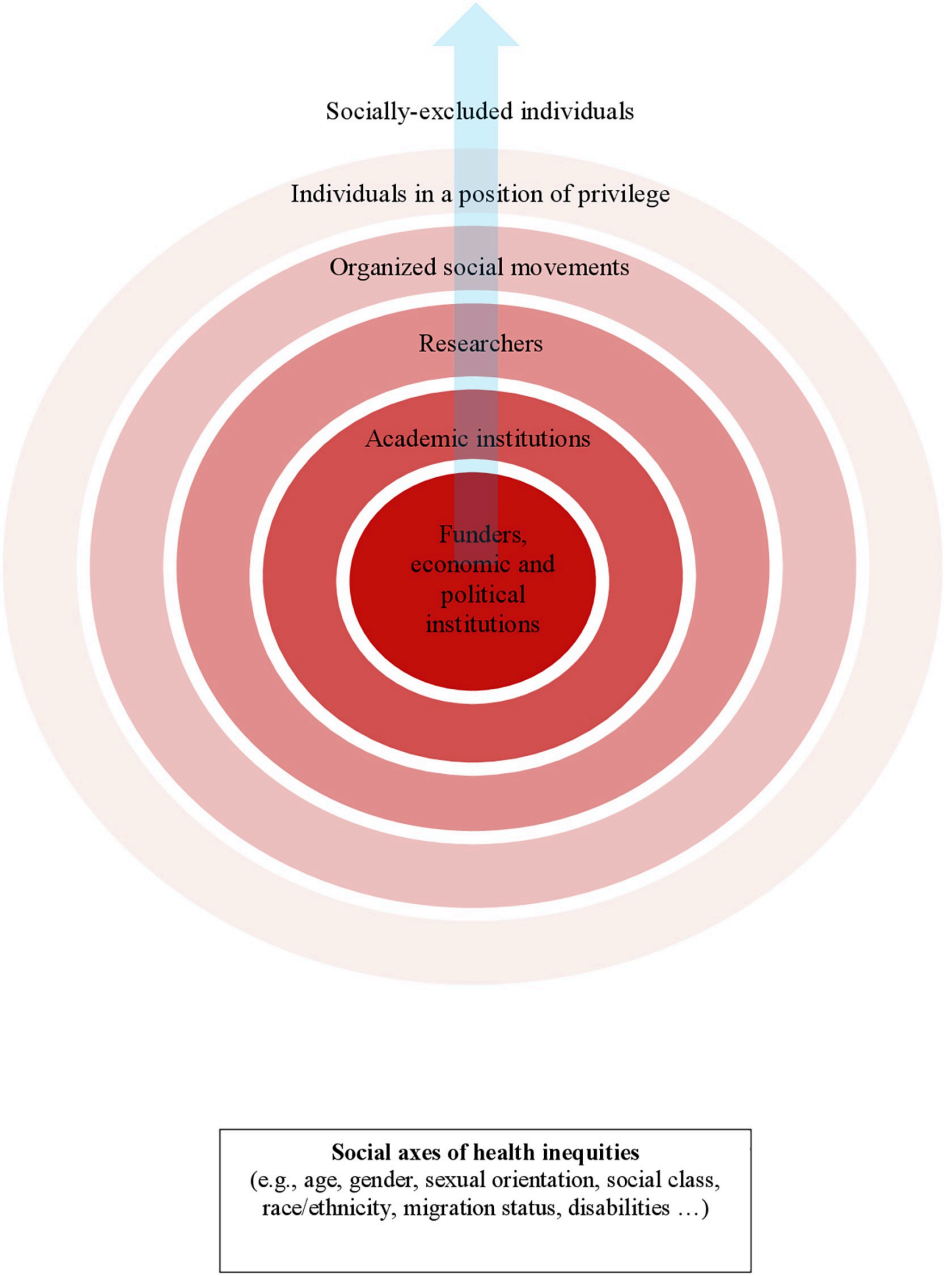
Power distribution of agents in public participation of scientific research.

Despite the differences between the two dominant discourses, they showed similarities regarding the distribution of power within PR in practice (Fig 2). According to our analysis, we have identified six different agents involved in PR with different degrees of power. We understand the power in this context as having the possibility to take part in relevant decisions concerning to the research project. Agents involved in public participation are ordered from those with more power to those with less power, from the inner to the outer circle. Power is primarily centred on research funders and economic and political institutions whereas socially excluded individuals are powerless in PR. Within the IPP discourse, the major role of the public was data collection and power was mainly held by agents within academic setting, as well as economic and political institutions that decide which projects are prioritised and receive funding. Conversely, the TPP discourse theoretically challenged hegemonic power held by institutions, and aimed to include the public in decision-making stages. In practice, however, power remained concentrated in funders, health systems and academic institutions, as researchers were constrained by the need for funding and academia’s norms.

As for the roles of the public, it is important to note that “the public” encompassed a wide variety of population groups that did not have the same opportunities to be involved in participatory processes. Eventually, only some agents of the public held had power and only to a certain extent: organised social groups (e.g., a patient organisation) and individuals in a position of power that belong to traditionally privileged population groups (e.g., individuals with high education). Conversely, socially excluded individuals included people from vulnerable and minority groups who do not belong to an organised social group (e.g., migrant in an irregular administrative situation) and, therefore, do not have the opportunity of being involved. In fact, we did not identify strategies aimed at the inclusion of socially excluded individuals in PR. Finally, we have also represented the intersection of the social axes of inequities (e.g., age, gender, social class, ethnicity …), which influences all agents transversally (e.g., power asymmetries related to the social axes of inequities within an institution, research team or a social organized group).

## 4. Discussion

This study presented the discursive positions on PR among individuals with experience in research and other participatory processes. We identified two dominant discourses, IPP and TPP, between experts in participatory process that can be placed at the two opposite ends of a continuum defined by the purpose and the (theoretical) power distribution in PR processes (see Fig 1). According to the IPP discourse, PR prioritises the purpose of improving research results. In addition, researchers and/or research institutions are the main responsible for decision-making in research. Conversely, the TPP discourse suggests that social changes are the main purpose of participatory processes and that decision-making within these processes must be shared between researchers and the public. Both discursive positions identified structural and ideological barriers to PR. Strategies targeting the inclusion of socially disadvantaged individuals or groups lacked in both positions.

Our findings are consistent with previous research on the values that are related to some PR framework [2, 24]. Values such as promoting empowerment, fundamental human rights, generating change or action, sharing power and decisions, among others are in line with TPP discourses identified in this research. For example, CBPR would be included within the TPP approach, since it is a transformative research paradigm that bridges the gap between science and practice through community engagement and social action to increase health equity [9]. Conversely, other values focus on the impact of public participation in research in terms of effectiveness, increasing quality and representativeness [2]. These are coherent with IPP discourses. Thus, the main purposes of public participation shifted from attaining social change and transforming to goals centred in institutional benefits (e.g., obtaining funding or improving scientific results). Although the outcomes of both IPP and TPP discourses are not necessarily opposed, the main goals of PR are remarkably different. In other words, although a PR process with an IPP approach may bring about changes and improvements for citizens, it is not focused on changing the social conditions of the population. Similarly, although a TPP approach might result in institutional benefits and better scientific results; this is not its ultimate goal.

Public participation was commonly framed around the benefits to research outputs (IPP) or the public (TPP). However, much less attention was paid to what public participation implies for and requires from researchers and institutions. For instance, training the public was identified as a requirement for participatory processes. However, training for researchers and institutions was given less importance. Assumptions that researchers and institutions are ready and equipped to lead public participation processes can be damaging to these processes. Reflexivity and critical questioning of such assumptions are needed so that the public can be successfully and meaningfully involved. Our recommendation would be to follow some guidelines that allows the research team to reflect on whether it is really willing and ready to undertake a PR [1].

We also identified ideological and structural barriers that need to be addressed to promote public participation [25]. Ideological barriers relate to how research or, more specifically, science is viewed by researchers. Indeed, researchers’ views about what is science shaped the role they assigned to citizens in PR and provide a glimpse about how PR or participatory processes are carried out. This is in line with the views of primary care health workers from Spain, which consider that leaving behind the hierarchical paradigm of science is essential to conduct PR [12]. Regarding structural barriers, both IPP and TPP discourses identified lack of funding and resources as the major barriers to carry out PR. However, ideological and structural barriers are ultimately interrelated, as funding agencies and research institutes have an ideology that translates into research grants and aids, thus reproducing this ideology within institutions. Funding schemes should be more flexible and available to all, including the public, so that participatory processes can become a reality both in academic and non-academic contexts. Flexibility is particularly relevant considering that what works well in one situation and at one point in time may be impossible in another [26]. However, careful attention should be paid to *tokenism*, as the inclusion of the public in research as a means to obtain funding for research appears to be rather common [25, 27]. Furthermore, tokenism could also hinder the value of public participation [25], especially when evaluations focus on outcomes and impacts instead of on the meaningfulness of the knowledge exchanges [28].

Evaluation seems to be an unresolved matter in PR. According to our results, evaluation is not carried out systematically, and PR is often perceived as too complex to be evaluated. To ensure that evaluation efforts are not hindered by this perception, moving forward pragmatically is fundamental [3]. Some informants mentioned personal and group reflexivity as evaluation strategies that provide valuable insights regarding the meaningfulness of the participatory process. Including such strategies might promote PR in health. In addition, time availability to carry out the PR should be taken into account, both from the perspective of the research teams and the public. This is especially relevant for conducting participatory process from a health equity approach, in order to include people with limited time availability, such as people who have to work long hours, as well as people with caregiver responsibilities [14].

Another important finding of our study is the researchers’ conception of the knowledge. Overall, IPP discourses seem to be mostly dominated by institution with consumerist ideologies, prioritising outcome’s over the process’ quality and seeing the public as “consumers” [29]. IPP is rather influenced by post-positivist or hegemonic biomedical approach, in which the researchers hold control over the research process [30]. On the other hand, TPP is closer to critical theories. Knowledge is a means for social emancipation and for promoting equity and social justice [30]. Thus, from TPP discourses, the concept of participation goes beyond recognizing population knowledge and questions power dynamics in the relationships between the researcher/researched and academia/the public [31]. Thus, TPP recalls the methodological proposals of Paulo Freire, who proposes listening deeply to the needs of the community, creating a dialogue for people to critically evaluate and delve into the problems of the community, promoting actions for people to change their living conditions, and permanent reflection on their actions [32]. In other words, it involves recognising lay knowledge, e.g. the role of people’s everyday life experiences [33]. Along these lines, it is interesting to note what feminist epistemologies propose, specifically Donna Haraway’s with “situated knowledge”. That is, to consider that the perception of any situation is always a matter of an embodied and situated subject that depends on geographical and historical perspective of people [34]. This makes it possible to assess personal knowledge and how it could influence in RP.

Power distribution is in fact a key element in public participation [24, 35]. Narratives on how power is distributed moved from those who considered that institutions and researchers should hold the power in PR (IPP) to those who questioned institutions and researchers’ rights to retain the power (TPP). However, in order not to oversimplify power structures, we should differentiate between the greater power held by institutions and the power held by the researcher team. Likewise, considering power inequities in participatory processes also means to review power differentials within the spheres of “the public”. In fact, even if TPP discourses included the need to tackle social inequities and promote inclusiveness, those generally excluded continued to be left out from public participation and privileged groups still dominate the majority of participatory processes. Therefore, “the public” cannot be understood as representative of the whole population or some groups, but represents subgroup of particular profiles of people who already control other socio-structural spaces due to gender, age, race/ethnicity, and migratory and socio-economic status. On the other hand, having flexibility in research is a more inclusive way of sharing power. This should be reflected in the openness of research teams to take on board what the citizens’ demand as priorities in health research. This refocuses the research agenda and embraces the power of the public. A clear example of this has been the social movements that emerged at the beginning of the HIV/AIDS pandemic and what is happening today with the COVID-19 pandemic. Currently, this means recognising the central role of emotion in social movements and in the biopolitics of any disease, as in the case of people with persistent symptoms (Long Covid) [36].

As a consequence, the underrepresentation of certain communities, such as minority ethnic groups might reproduce systems of social inequities within participatory processes [37, 38]. Moreover, even if these groups are included, opportunities to participate are not equally distributed among these groups since individuals collaborating in socially organised groups are more likely to be involved than the others. Attention to social inequities and the adoption of intersectional approaches might be useful, so that participatory processes are more approachable to those groups and individuals who have been historically invisible and unheard in PR [38]. Building non-institutionalised spaces and promoting horizontal relationships considering different contexts would also be crucial, especially to effectively involve marginalised communities and considering different contexts [26, 39]. This brings into focus the creation of ’participatory spaces’ to improve understanding of the factors and dynamics that shape decision-making between stakeholders from different backgrounds and with different perspectives, such as health researchers and patients/community members [40]. On the other hand, it must be noted that to date the majority of publications in PR are from high-income countries, which influences the type of evidence available [26]. For example, is being produced mainly in the US and UK, followed by research in Australia, Germany and Canada [6]. Despite the fact that Latin American countries, for example, have extensive experience in PR [32]. These results could be understood from a postcolonial approach, in which scientific knowledge has been produced predominantly in Western epistemologies [41].

Children and adolescents are another group population underrepresented in participatory processes, even though research projects address often issues that directly concern them, especially in more vulnerable groups [42]. On the other hand, it is crucial to incorporate different stakeholders during the research in order to have a greater social and health impact [43]. Therefore, it is important to prioritise the inclusion of all social groups, based on the idea they will be capable of making relevant contributions if given the same opportunity to speak [44]. In summary, achieving truly inclusive participatory processes is essential to, actively avoid the reproduction of social inequities in health [16, 45].

### 4.1 Limitations and strengths

The most relevant limitation of this study is that we did not include the public in the research team. Nevertheless, the research team undertook a deep process of reflection on their own research practices when designing the project. We continually questioned our position as researchers and when we assumed the role of participant. After some reflection as a research team, we have realized how conventional structures of research are also embedded within our own research team. We thought of this reflection as another finding and point of discussion. On the other hand, the study provides relevant knowledge on participatory processes in health research, which can be very useful in our context. We believe our findings will contribute to promote co-production of knowledge through the participation of different stakeholders including individuals socially disadvantaged. Another strength of this study was the use of a CDA methodology, which was useful for a more in-depth and critical theoretical analysis.

## 5. Conclusions

Understanding discourses on public participation from people self-considered as experienced in PR and participatory processes can help us understand the conceptualisations of PR, and how these are performed. We identified two dominant discourses of PR, instrumental public participation (IPP) and transformative public participation (TPP). The central elements of these two discourses were the main purpose of public involvement (institutional benefits vs social change) and the power distribution within participatory processes (research decision-making centred in institutions vs shared with the public). Despite the differences between these discursive positions, we found that power remains in economic resources primarily centred on research funders and economic/political institutions that are embedded in an ideology about knowledge. However, in both discourses (and actions) socially excluded individuals are powerless in PR. For instance, the discourses positions stated that they do not carry out specific strategies to include people or groups that suffer greater socially disadvantaged. This suggests that within the same group there are differences in the opportunities for participation. Within vulnerable groups, individuals with higher level of agency and social support are more likely to be involved in participatory processes.

Benefits of public participation need to be framed, not only from an institutional perspective, but from the public’s interests. In order to do this, it is especially relevant to involve socially excluded and marginalised communities in participatory processes, as well as, to consider the importance of lay knowledge of people. Therefore, political and institutional commitments are needed to bring public participation’s into practice. A shift in the scientific approach to knowledge, along with time and resources, are required to move towards a more balanced power distribution in the processes involving the public and their health.
